# Case Report: Giant Pelvic Cystic Appearance—An Unusual Feature of Uterine Cervical Adenocarcinoma

**DOI:** 10.3389/fsurg.2022.841255

**Published:** 2022-03-09

**Authors:** Yongxin Wang, Xue Shan, Man Li, Ying Yue

**Affiliations:** ^1^Department of Gynecologic Oncology, The First Hospital of Jilin University, Changchun, China; ^2^Department of Cardiac Surgery, The First Hospital of Jilin University, Changchun, China

**Keywords:** adenocarcinoma, cervical cystic tumor, uterine cervical neoplasms, pelvic cystic tumor, cervical cyst

## Abstract

Cervical adenocarcinoma can present as a solid, mixed solid, cystic, or multiple cystic cervical mass in the endocervical canal. In this report, we present an extremely rare case of cervical adenocarcinoma with giant cystic lesions. A 37-year-old Chinese woman with a regular menstrual cycle presented to her local doctor complaining of mild abdominal distension. Abdominal ultrasonography suggested an ovarian cyst, whose mean diameter increased from 3 to 8 cm in 3 months. Thereafter, she was referred to our hospital. She had no abnormal vaginal bleeding or discharge. Transvaginal ultrasonography revealed a 95 × 80 mm cyst below the back of the uterus. Computed tomography revealed a 9.8 × 8.5 cm multilocular cyst between the cervix and right ovary. Human papillomavirus (HPV)-E6 and -E7 mRNA tests revealed HPV-16 positivity. The thin-layer, liquid-based cytological test of the cervix showed negative results. No tumor lesions were observed on the cervical biopsy histopathology. The lesion was misdiagnosed as an ovarian cyst prior to the surgery. Intraoperatively, a cyst of the size of a child's head was observed extending from the low posterior wall of the uterus to the posterior lip of the cervix, and the cervical cysts were resected. Histological examination revealed cervical adenocarcinoma. Subsequently, she underwent extensive hysterectomy, bilateral salpingectomy, and pelvic lymphadenectomy. The final diagnosis was stage IB3 cervical adenocarcinoma. After 21 months of follow-up, no clinical or radiological evidence of recurrence has been found.

## Introduction

Cervical cancer is the fourth most frequent cancer among women globally. As of 2018, China and India contributed to more than one-third of the global cervical burden (1). Histologically, squamous cell carcinoma of the cervix (SCCC) accounts for ~75% of all cervical cancers, while adenocarcinoma of the cervix (ADCC), accounts for ~15% (2). ADCC incidence continues to increase with a decrease in the incidence of SCC as a result of improved screening programs (3). The MRI findings of a variety of cervical tumors can be grossly classified into five patterns: type 1, multi-cystic, or solid and cystic pattern; type 2, exophytic villous pattern; type 3, expansile solid pattern; type 4, invasive solid pattern; and type 5, diffuse infiltrative pattern. Some of the tumors mainly show any one of these typical imaging patterns, while others show mixed patterns. The histopathological types can be classified into five types (4). Adenocarcinomas usually appear as types 1, 4, and 5, whereas squamous cell carcinomas, which can be necrotic, usually do not have defined cystic spaces (4, 5). In this report, we describe an extremely rare case of cervical adenocarcinoma with giant cystic lesions. The patient had no obvious clinical symptoms and was misdiagnosed preoperatively.

## Case Description

**Figure 1:**
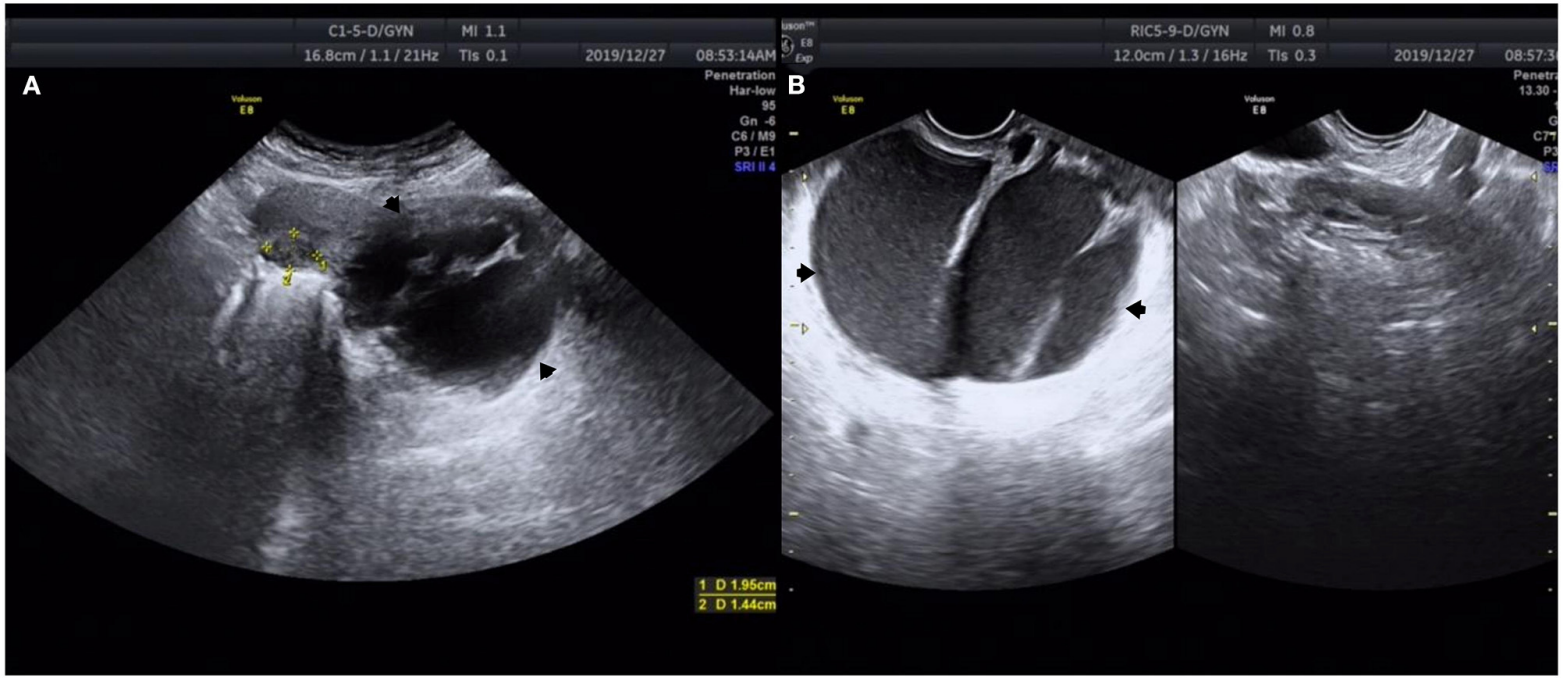
Ultrasound images of a 37-year-old woman with cervical adenocarcinoma. **(A)** Transabdominal ultrasound shows a 95 × 80 mm cyst below the back of the uterine cervix (black arrow). **(B)** Transvaginal ultrasound shows that the cyst fluid was viscous and there was a large separation in the cyst (black arrow). *Measurement of uterine leiomyoma markers.

**Figure 2:**
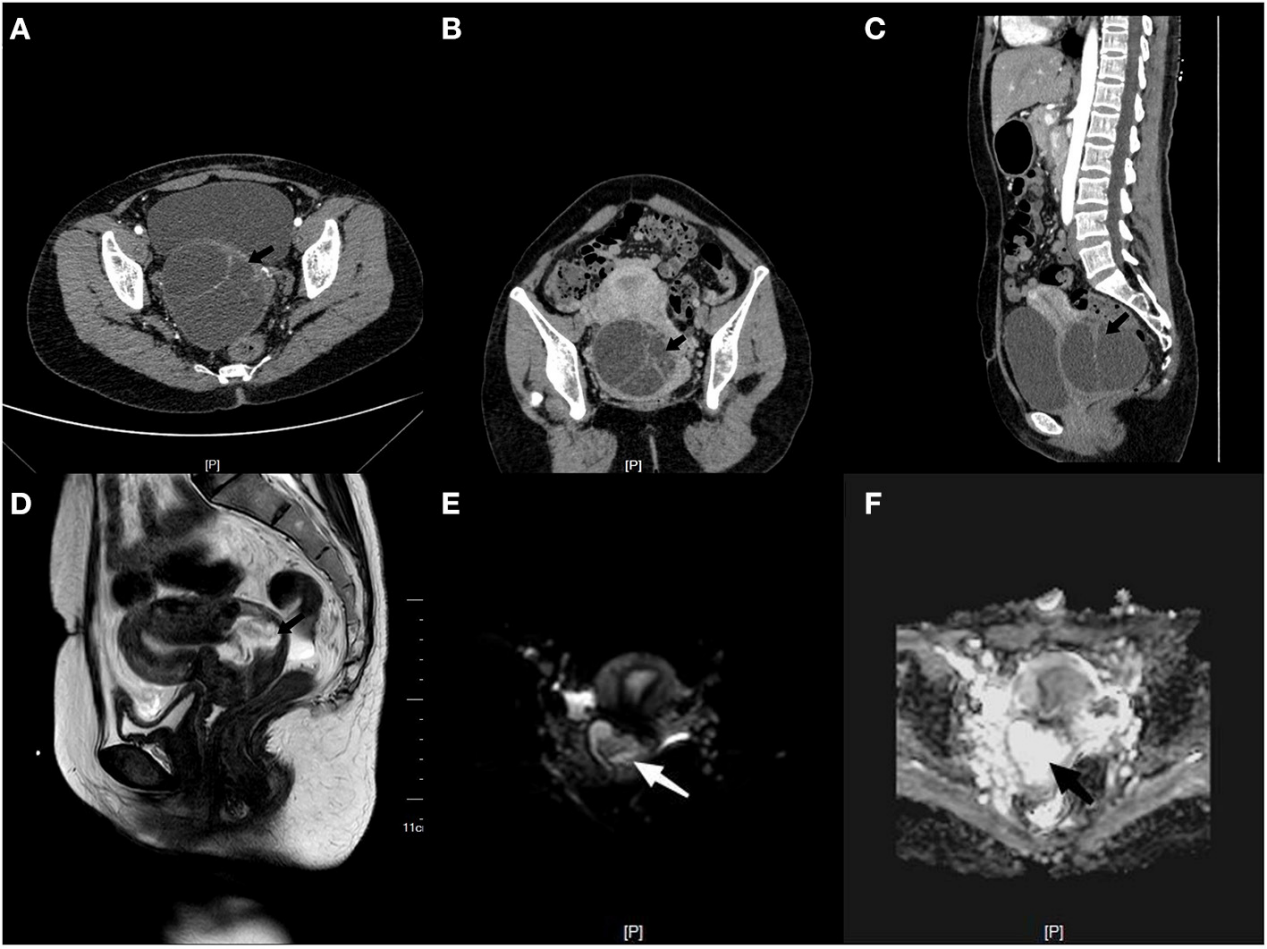
Computed tomography showing the irregular shape of uterus. **(A)** There is a large abnormal density of multilocular cystic shadow, which is ~8.8 × 8.5 cm in size (black arrow). **(B)** There is an irregular separation in the cyst, and there is a close relationship between the uterus, cervix, and right appendix (black arrow). **(C)** Three-dimensional computed tomography reconstruction showing the cyst from below the posterior wall of the uterus to the posterior lip of the cervix (black arrow). **(D)** After cyst resection, additional MR examination showed irregular shape of posterior lip of cervix, size 4.1 × 2.3 × 4.7 cm, inhomogeneous low signal on T1WI (black arrow). **(E)** Postoperative lesions under diffusion weighted imaging (white arrow). **(F)** Postoperative lesions in ADC images (black arrow).

**Figure 3:**
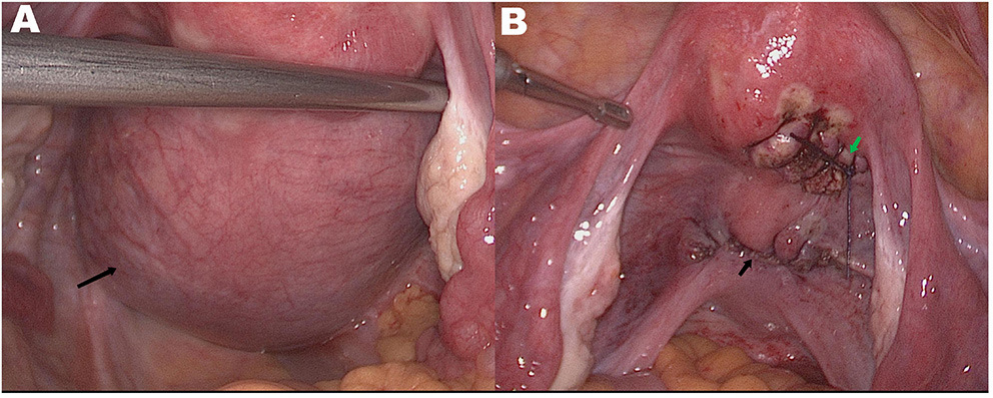
**(A)** Preoperatively bilateral ovaries and fallopian tubes are normal. A smooth and intact cyst surface with a diameter of 9 cm can be seen from below the posterior wall of the uterus to the posterior lip of the cervix (black arrow). **(B)** Uterine and cervical morphology returned to normal after operation. Surgical wound after myomectomy (green arrow) and after cervical cyst resection (black arrow).

**Figure 4:**
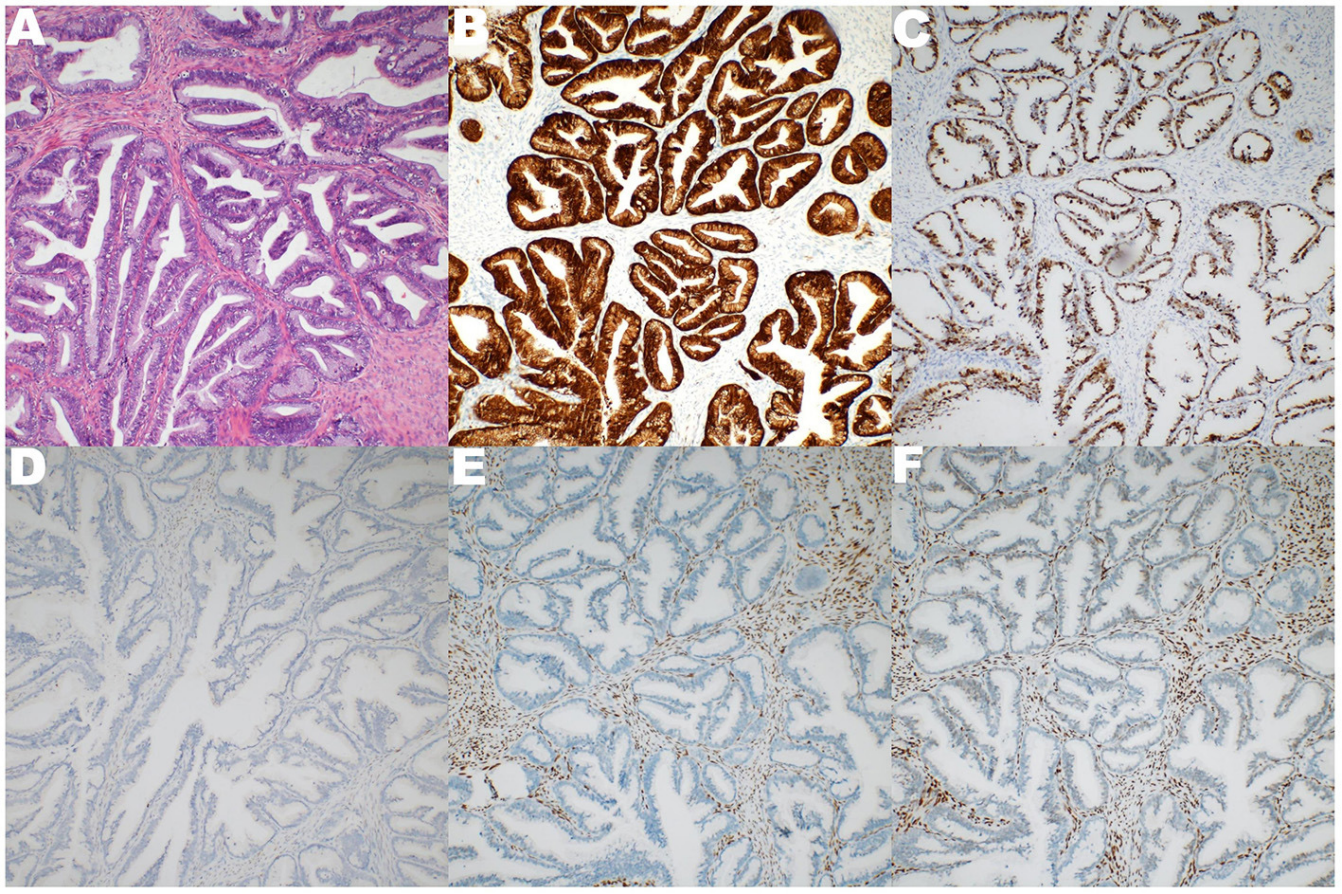
The specimen representing broken cystic wall-like tissue, 5 × 4.5 × 2 cm in size. Pathological diagnosis is HPV-associated adenocarcinoma (HPVA). **(A)** The components of carcinoma were lobular, tubular and villous in smooth muscle and fibrous tissue of cystic wall. **(B)** Immunohistochemical P16 positive. **(C)** Immunohistochemical Ki-67 50% positive. **(D)** Immunohistochemical WT-1 negative. **(E)** Immunohistochemical ER negative. **(F)** Immunohistochemical PR negative.

A 37-year-old Chinese woman (gravida 4, para 1) with a regular menstrual cycle presented to her local doctor with a complaint of mild abdominal distension. Abdominal ultrasonography suggested an ovarian cyst with an increasing mean diameter from 3 to 8 cm in 3 months. Thereafter, she was referred to our hospital. She had no abnormal vaginal bleeding or discharge. She had a history of cesarean section 10 years ago and uterine myoma (diameter: ~2 cm) 3 years ago. Transvaginal ultrasound showed that the size of the uterus was 64 × 36 mm, and fibroids measuring 20 × 14 mm were found in the posterior wall. A 95 × 80 mm cyst was observed below the back of the uterus. The cyst had multiple partitions with viscous internal fluid. The left ovary was normal, and the right ovary was not visible (Figure 1). Three-phase enhanced whole abdomen computed tomography (CT) showed an 8.8 × 8.5 cm multilocular cyst between the cervix and right ovary (Figure 2). The radiologists considered that the cyst might have originated from the cervix, and no enhancement was observed around the cyst wall. Serum cancer antigen (CA)125, CA199, and carcinoembryonic antigen levels were within the normal range. Physical examination revealed that the exocervix was small and flat, with a very deep position due to the traction of adhesion after cesarean section. A large cyst was palpable at the Douglas fossa, and the uterus moved with the cyst. The appearance of the cervix on speculum examination was unremarkable. Human papillomavirus (HPV)-E6 and E7 mRNA tests showed HPV-16 positivity. A thin-layer liquid-based cytological test (TCT) of the cervix revealed negative findings. The patient with a high-risk HPV infection was diagnosed with no tumor lesions on cervical biopsy histopathology. A giant cyst of the cervix is rare, and although the CT report was assessed without detailed view of CT images, owing to the patient's long history of pelvic ultrasonography, the initial diagnosis was a right ovarian cyst and uterine myoma. The patient underwent laparoscopic surgery the day after admission. Intraoperatively, the bilateral ovaries and fallopian tubes were normal. A cyst measuring around a child's head (Figure 3) was observed extending from the lower posterior wall of the uterus to the posterior lip of the cervix. The cyst surface was smooth and intact. A myoma with a diameter of 1.5 cm in the left posterior wall was observed. Since the cervical cyst did not show malignant growth patterns, we decided to strip the cyst and excise the uterine myoma. We first removed the hysteromyoma and sewed the muscle wall, and then cut the serous layer on the cyst surface layer by layer to expose the thin cyst wall. The boundary between the cyst and the surrounding cervical tissue was unclear. During stripping, the cyst ruptured, and the transparent and viscous cyst fluid flowed out. A few partitions were observed in the cyst. The root of the cyst was located at the posterior lip of the cervix and reached the internal orifice of the cervix. To completely remove the cyst tissue, we cut off the upper part of the cyst tissue in the abdominal cavity, and the root of the cyst was removed from the cervical canal through the vagina. The sample was sent for frozen section analysis, and abnormal glands were observed in the cervical tissues, which could be malignant. The uterus was sutured, and peritoneal lavage fluid was left for cytological examination. Histological examination revealed a cervical adenocarcinoma. The tumor invaded the smooth muscle fiber stroma without vessels and nerves. The uterine tumor was a leiomyoma. The carcinoma components comprised lobular, tubular, and villous growth patterns distributed in the capsule wall, smooth muscle, and fibrous tissue. The immunohistochemical staining results showed PAX-8, P16, and Ki-67 (50%) positivity and vimentin, ER, PR, and WT-1 negativity, further verifying the diagnosis of cervical adenocarcinoma (Figure 4). Pathological examination was performed at the Pathology Center of Peking University Third Hospital, and the results were consistent with those of HPV infection-related cervical adenocarcinoma. Cytological examination of peritoneal lavage fluid revealed no tumor cells. Pelvic magnetic resonance imaging revealed a suspicious lesion in the cervix. However, no ligament involvement was observed, except in the cervix (Figure 2). Her serum squamous cell carcinoma-related antigen (SCC) level was normal. After further discussion with the oncology team, exploratory laparotomy was performed with the intent of extensive hysterectomy, bilateral salpingectomy, and pelvic lymphadenectomy. After frozen sections of bilateral ovarian biopsies were found to be negative, we reserved the bilateral ovaries. The overall histological and histochemical features of the specimen obtained during laparotomy revealed no adenocarcinoma components in the resected specimen. No tumor was found in the uterus, bilateral fallopian tubes, or pelvic lymph nodes. Based on the two pathological results and cyst size, the final diagnosis was stage IB3 cervical adenocarcinoma according to the International Federation of Gynecology and Obstetrics (FIGO) staging system. Since all the tumors were located in the cervical cyst, the depth of invasion could not be determined. However, the cyst was 9 cm in diameter, protruded into the abdominal cavity, and ruptured intraoperatively. After a detailed discussion by gynecologists, medical oncologists, and radiologists, postoperative radiotherapy and chemotherapy were recommended to prevent recurrence. However, the patient refused radiotherapy because of the fear of ovarian dysfunction. The treatment included intravenous paclitaxel at 175 mg/m^2^ administered over 3 h, followed by carboplatin at a dose giving an area under the time–concentration curve of 5 mg × min/mL (based on the calculated creatinine clearance) diluted in 500 mL of 5% glucose administered over 1 h. Both drugs were administered once every 3 weeks and two courses were administered. Follow-up visits every 3 months consisted of clinical assessment for any signs of recurrence, together with the HPV E6 and E7 mRNA and TCT at the vaginal stumps, CA125 test, SCC assay, and chest and pelvic CT. After 21 months of follow-up, no clinical or radiological evidence of recurrence was found.

## Discussion

In this report, we have described an extremely rare case of HPV-associated adenocarcinoma (HPVA) with a large cystic lesion (diameter: ~10 cm). In contrast, most of the previously reported cervical cysts were multiple minor cysts of the cervix, mostly benign, and common in adenomas.

Adenoma malignum (6–9) is also known as minimal deviation carcinoma (MDA). Although MDA is rare in clinical practice, it is easy to identify because of its typical imaging features. Manifestations of multiple cystic lesions in the cervix with solid components are not commonly associated with HPV. In our case, the patient was HPV positive, with normal cervical biopsy and TCT findings; only a huge cyst, which was different from previously reported malignant cervical lesions, was found in the cervix. Therefore, the possibility of malignancy was ignored before surgery. The presence of large cervical cysts is a rare clinical finding. They compress normal cervical tissues and change their normal cervical morphology. However, due to the high incidence of ovarian cysts, they are easily misdiagnosed as adnexal cysts. A retrospective analysis of the ultrasound image of a patient showed that the cyst position was low, occupying the position of the posterior lip of the cervix. The uterine and cervical contours were incomplete, and the cyst was thickly separated and filled with viscous cyst fluid. CT examination is not very useful for determining the location of cysts in a cross-sectional view. It can be observed on the sagittal plane by three-dimensional reconstruction, which is more conducive to diagnosis (Figure 2). Since the diagnosis of cervical cysts was not considered before surgery, preoperative magnetic resonance (MR) examination images were lacking. After cyst resection, additional MRI showed no other lesions in the posterior cervical lip. Most reported cases of cervical cysts are multiple small cysts <2 cm in diameter. Only one case of cervical minimal deviation carcinoma with a diameter of 10 cm has been reported in Japan (7). Another cervical minimal deviation carcinoma with a diameter of 6 cm was reported in India (10). Minimal deviation adenocarcinoma of mucinous type was considered part of the spectrum of gastric-type adenocarcinoma, which is a non-HPVA. After diagnosis in two large pathological centers, the patient was considered to have HPVA, and the positive immunohistochemical P16 and HPV E6 and E7 mRNA were also consistent with this diagnosis. The prognosis of HPVA is better than that of non-HPV cervical adenocarcinoma (8). There is no standard FIGO staging method for cervical adenocarcinoma of large cysts. However, since the diameter was 10 cm and the tumor infiltrated into the stroma and muscle layer with no parametrial infiltration, it was diagnosed as a stage IB3 tumor. Previous randomized controlled trials have shown that laparoscopic cervical cancer surgery has a higher risk of recurrence than open surgery (11). The patient underwent laparoscopic surgery for the first time, and during the procedure the cyst ruptured, and the cyst fluid leaked into the abdominal cavity. There may be two courses of chemotherapy for patients to reduce the risk of tumor recurrence. Although the patient refused radiotherapy, no relapse was observed at the 2-year follow-up. The mRNA test was evaluated as a biomarker for persistent or recurrent disease in women with CIN2+. Follow-up with mRNA tests shows high specificity for early detection of the risk of residual or recurrent cervical lesions (12). During the follow-up, HPV E6 and E7 mRNA and TCT at the vaginal stumps were negative persistently.

Due to their rare occurrence, doctors have insufficient understanding of large cervical cysts. Through this case report, we hope to enhance the identification of cervical cysts. First, when large pelvic cysts are found, the possibility of ovarian and tubal cysts only should not be considered. In cases where the cyst position is low, the cyst is in close proximity to the cervix, and the ultrasound examination does not show a complete cervical morphology, the presence of cervical cysts should be considered. Appropriate imaging examinations can help prevent unnecessary surgery before histopathologic proof of malignancy and can facilitate prompt and accurate diagnosis and treatment. Enhanced CT cannot only show the cervical cyst, but also accurately judge the staging of tumor when the lesion is malignant. However, due to the low resolution of CT image, the relationship between lesions and surrounding tissues cannot be well-distinguished. It is not easy to distinguish the source of lesions when the cyst is huge, and the pelvic tissue is squeezed. CT post-processing technology can make up for this deficiency to some extent. MRI can clearly show the relationship between cervical cyst and surrounding tissues. It can also be observed whether there is periuterine or interstitial infiltration, used to indicate benign and malignant lesions. Second, even if the size and morphology of the cervix under endoscopy appear normal, TCT and cervical biopsy are negative, high-risk (HR) HPV-positive or -negative, solitary or multiple cervical cysts have the potential of developing into malignant adenocarcinoma, especially with rapid growth. For these patients, cervical biopsy along with cervical curettage should be performed. If the results are negative, cervical conization should be considered to reduce the missed diagnosis of cervical malignant tumors and high-grade cervical dysplasia (13). A multicenter retrospective study showed that patients who had a preoperative diagnosis through conization had a significantly lower rate of recurrence than those who underwent cervical biopsy (14). Third, Because cervical adenocarcinoma is more likely to recur than squamous-cell carcinoma, patients should be followed up regularly. Italy reported a case of cervical adenocarcinoma stage IIIB which showed rapid and diffuse disease progression from the site of the lesion to the pelvic bones occurred 2 years after radical surgery (15). Markers such as CEA, SCC-Ag, and CD44 can be used for early diagnosis, evaluation, and monitoring of therapeutic interventions to improve diagnosis and treatment (16).

## Data Availability Statement

The original contributions presented in the study are included in the article/supplementary materials, further inquiries can be directed to the corresponding author/s.

## Ethics Statement

Written informed consent was obtained from the patient for publication of potentially identifiable images or data included in this manuscript.

## Author Contributions

YW and XS drafted the manuscript. YW and YY designed the clinical treatment for the patients and performed the clinical treatment for the patients. YW, ML, and XS provided comments and edited the manuscript to become the final version for submission. All authors contributed to the manuscript and approved the submitted version.

## Conflict of Interest

The authors declare that the research was conducted in the absence of any commercial or financial relationships that could be construed as a potential conflict of interest.

## Publisher's Note

All claims expressed in this article are solely those of the authors and do not necessarily represent those of their affiliated organizations, or those of the publisher, the editors and the reviewers. Any product that may be evaluated in this article, or claim that may be made by its manufacturer, is not guaranteed or endorsed by the publisher.
